# Modelling habitat suitability in Jordan for the cutaneous leishmaniasis vector (*Phlebotomus papatasi)* using multicriteria decision analysis

**DOI:** 10.1371/journal.pntd.0008852

**Published:** 2020-11-23

**Authors:** Emi A. Takahashi, Lina Masoud, Rami Mukbel, Javier Guitian, Kim B. Stevens

**Affiliations:** 1 Royal Veterinary College, Hawkshead Lane, Hatfield, Hertfordshire, United Kingdom; 2 Department of Basic Medical Veterinary Science, Faculty of Veterinary Medicine, Jordan University of Science and Technology, Irbid, Jordan; Universidade do Estado do Rio de Janeiro, BRAZIL

## Abstract

Cutaneous leishmaniasis (CL) is a zoonotic vector-borne neglected tropical disease transmitted by female *Phlebotomine* sand flies. It is distributed globally but a large proportion of cases (70–75%) are found in just ten countries. CL is endemic in Jordan yet there is a lack of robust entomological data and true reporting status is unknown. This study aimed to map habitat suitability of the main CL vector, *Phlebotomus papatasi*, in Jordan as a proxy for CL risk distribution to (i) identify areas potentially at risk of CL and (ii) estimate the human population at risk of CL. A literature review identified potential environmental determinants for *P*. *papatasi* occurrence including temperature, humidity, precipitation, vegetation, wind speed, presence of human households and presence of the fat sand rat. Each predictor variable was (a) mapped; (b) standardized to a common size, resolution and scale using fuzzy membership functions; (c) assigned a weight using the analytical hierarchy process (AHP); and (d) included within a multicriteria decision analysis (MCDA) model to produce monthly maps illustrating the predicted habitat suitability (between 0 and 1) for *P*. *papatasi* in Jordan. Suitability increased over the summer months and was generally highest in the north-western regions of the country and along the Jordan Valley, areas which largely coincided with highly populated parts of the country, including areas where Syrian refugee camps are located. Habitat suitability in Jordan for the main CL vector—*P*. *papatasi—*was heterogeneous over both space and time. Suitable areas for *P*. *papatasi* coincided with highly populated areas of Jordan which suggests that the targeted implementation of control and surveillance strategies in defined areas such as those with very high CL vector suitability (>0.9 suitability) would focus only on 3.42% of the country’s total geographic area, whilst still including a substantial proportion of the population at risk: estimates range from 72% (European Commission’s Global Human Settlement population grid) to 89% (Gridded Population of the World) depending on the human population density data used. Therefore, high impact public health interventions could be achieved within a reduced spatial target, thus maximizing the efficient use of resources.

## Author summary

Cutaneous leishmaniasis (CL) is a globally distributed zoonotic vector-borne neglected tropical disease transmitted by female sandflies. The aim of this study was to estimate habitat suitability of the CL-transmitting sandfly in Jordan using spatial data on variables that influence the sandflies’ presence and survival including temperature, humidity, precipitation, vegetation, wind speed, presence of human households and presence of the fat sand rat. Once high-risk areas were identified, the number of people at risk of infection could be estimated. Sandfly habitat suitability increased over the summer months and was generally highest in the north-western parts of the country and along the Jordan Valley, coinciding with highly populated regions, including areas containing Syrian refugee camps. These results suggest the need for targeted surveillance and disease control efforts.

## Introduction

### Cutaneous leishmaniasis background and epidemiology

Cutaneous leishmaniasis (CL) is one of the 17 neglected tropical diseases (NTDs) identified by the World Health Organization (WHO) during the 10^th^ meeting of the Strategic and Technical Advisory Group of Neglected Tropical Diseases in 2017 [1]. Despite causing a large disease burden among infectious diseases–Alvar *et al*. [2] estimated 1.2 million new cases of CL globally per year—CL is rarely included in tropical disease priorities. This is possibly due to its complex epidemiology and ecology, lack of current incidence data, lack of readily available tools for case management, and challenging treatment [2]. Cases are focally concentrated with approximately 70–75% of the estimated global CL incidence occurring in just ten countries namely Afghanistan, Algeria, Brazil, Colombia, Costa Rica, Ethiopia, Iran, Peru, Sudan and Syria [2].

CL is caused by an obligate intracellular protozoan from the genus *Leishmania*, which is spread through the bite of infected female *Phlebotomine* sandflies and is maintained by mammalian hosts [3,4]. There are more than 800 named sandfly species distributed globally of which 78 are currently known to be vectors for *Leishmania* spp. [5]. CL can either be anthroponotic if the natural reservoir of the parasite is human, or zoonotic if there is a suitable animal reservoir, of which the main ones are canids (e.g. dogs, foxes, jackals and wolves) and rodents (e.g gerbils, jirds and fat sand rats) [6]. There are numerous geographically specific *Leishmania*-sandfly combinations able to transmit CL, and 98 countries from five continents have reported endemic transmission [2,4,7]. However, some countries are considered endemic despite not reporting any human cases, due to detection of the *Leishmania* parasite in animal reservoir populations. For example *L*. *major* has frequently been isolated from gerbils in Mongolia but no human cases have been reported [2]. The fat sand rat (*Psammomys obesus*) is considered to be the main animal reservoir for *L*. *major* [8–10], with one study in Tunisia reporting up to 70% of trapped *P*. *obesus* testing positive for *L*. *major* [11]. *P*. *obesus* has a close ecological relationship with the sandfly *Phlebotomus papatasi* since the rodent’s burrow provides sandflies with a moist and protected environment that is crucial for survival and reproduction. They offer adult sandflies a stable blood meal source, and larvae with necessary organic debris on which to feed and as a result *P*. *papatasi* is frequently found nearby *P*. *obesus* burrows [12–15].

### Cutaneous leishmaniasis in Jordan: distribution and prevalence

CL is endemic in Jordan with the first case reported in 1929 [3]. It is mainly caused by *L*. *major* and transmitted by the *P*. *papatasi* sandfly [10]. There have been some sporadic CL cases caused by *L*. *tropica* in the northern border region, but it is relatively uncommon compared to *L*. *major* [16–18]. Although CL outbreaks have been regularly reported in different parts of the country [18–23], areas close to the Jordan Valley and the Dead Sea are considered the main endemic foci. For example, regions such as Swaimeh presented hyperendemicity characterized by 100% positive skin tests in individuals over the age of five in 1992 [21]. Moreover, detection of CL animal reservoirs like *P*. *obesus* has taken place in areas close to the Jordan Valley and the Dead Sea [15,24,25], which coincided with locations where CL cases have historically been reported [18,19,21–23].

In Jordan, CL is a notifiable disease and treatment is free. There is no national control programme and surveillance consists of passive case detection with weekly reports by physicians from local health departments (governorate level) to the Directorate for Disease Control, Ministry of Health (MoH) [26]. Although cases of CL have been reported annually, fluctuations in numbers may potentially represent variability in reporting capacity as severe under-reporting was described by Mosleh *et al*. [26] who detected a 47-fold difference between reported cases and those identified through active case detection.

Over the past few years, Jordan has witnessed a significant influx of Syrian refugees. Large-scale human displacement and migration from non-endemic areas to areas with active CL transmission, or *vice versa*, are of particular concern regarding the association between conflict-terror and CL outbreaks [27]. This can result in more first-time exposure to CL, or the introduction of CL into new areas through susceptible sandfly vectors. Large-scale cross-border migration from Syria has taken place towards neighbouring countries such as Turkey, Lebanon, Jordan and Iraq [28], and CL cases have been reported in both refugee and non-refugee populations in Lebanon and Turkey [29,30]. Refugee camps and temporary settlements provide ideal conditions for enhanced transmission due to overcrowding, inadequate sanitation, waste disposal and inadequate housing, all conditions which characterised past CL outbreaks in refugee camps in Lebanon and Afghanistan [29,31]. Syrian refugee camps in Jordan are therefore at risk of CL outbreaks and should be considered within the scope of CL vector habitat suitability.

### Mapping CL distribution in Jordan

In 2014 the WHO prepared the ‘*Framework for action on cutaneous leishmaniasis in the Eastern Mediterranean Region 2014–2018’*. It was developed in consultation with regional member states and CL experts, aiming to strengthen surveillance, case management, disease prevention, capacity-building and research to reduce the burden of CL in the region. Proposed country-level actions included, amongst others: (i) the implementation of geographical information systems (GIS) to map and identify risk factors and areas of transmission risk, and (ii) the assessment of sandfly ecology to develop low environmental impact vector prevention and control plans [32].

Risk maps are valuable tools in public health that assist with decision-making by providing visual cues with which to prioritize and target prevention and control measures. The development of risk maps for vector borne diseases (including CL) often relies on vector exposure data instead of data on cases of infection to map risk. A primary advantage of using vector data over data on cases of infection includes the use of higher resolution data that are not restricted by patient data protection and anonymity. In addition, it overcomes the limitations associated with under-reporting, and uses accurate disease exposure locations which cannot be captured by epidemiological data as these generally include patient domicile or reporting healthcare centre as locations [33]. Vector exposure risk maps have been developed using different GIS-based statistical models to estimate vector presence or abundance within a specific geographical area. Spatial models can be based on the spatial dependence of vector data (interpolation), or on the links between vector data and underlying environmental or socioeconomic variables (extrapolation). Spatial model outputs construct risk maps on a continuous surface and thus include information in areas that would be missing from surveillance programmes and epidemiological data [33,34]. Finally, the inclusion of population and demographic data can facilitate the estimation of the population at risk of vector exposure, which can be used as a proxy for risk of disease [35].

Spatial models previously built to explore the distribution and abundance of CL vectors generally comprised data-driven statistical models including general and generalized linear models [36–38], cluster analysis [39], principal component analysis [40] or ecological niche modelling [41–46]. However, these methods all require data–hence data-driven–from which to quantify associations between species’ distribution and predictors, and therefore cannot be used when robust species distribution data are lacking, as in this instance.

In situations where georeferenced data describing a species distribution in environmental space with reference to a set of explanatory variables are not available, methods such as multicriteria decision analysis (MCDA), that use knowledge of the species distribution as model inputs–hence knowledge-driven–provide a valid alternative approach for generating habitat suitability maps that can be used to define the estimated distribution of a vector species [34] and inform targeted surveillance and control measures.

MCDA applies knowledge-driven rules through the implementation of Saaty’s [47] analytical hierarchy process (AHP) and predictor-based fuzzy memberships to identify areas suitable for the occurrence of a vector or disease [48]. AHP is a framework which allows multi-layered decisions to be made—the comparative credible weighting of interwoven predictors, or criteria, with respect to species’ habitat suitability—by reducing complex, multi-criteria comparisons (temperature versus rainfall versus humidity) to multiple simple pairwise comparisons (temperature versus rainfall; rainfall versus humidity; humidity versus temperature) to estimate the overall contribution of each predictor to habitat suitability. Advantages of using MCDA over data-driven modelling methods include its participatory element enabling the incorporation of multiple stakeholder perspectives and qualitative information into an unambiguous and transparent decision-making framework [34]. Furthermore, by not requiring the collection of data, this method is inexpensive and not subject to some of the traditional limitations and biases related to data collection methods [34,35]. Despite the increasing use of spatial MCDA in public health over the past decade, its application to vector-borne diseases remains limited [35,49–53]. However, in Jordan, where few studies have investigate the distribution of the CL vector, *P*. *papatasi* [15,24,54], and where there is a lack of robust national entomological data to feed such investigations, a knowledge-driven approach such as MCDA is an obvious and valid alternative for creating accurate risk maps in the absence of species’ distribution data [34,55,56].

Although MCDA has been used to model the distribution of CL in north-eastern Iran [57], to the authors’ knowledge this is the first study to use MCDA in the production of habitat suitability maps for leishmaniasis vector exposure, which could potentially guide and target interventions to minimise the burden of CL in Jordan.

### Study aims and objectives

The aim of this study was to map habitat suitability of the main CL vector *P*. *papatasi* in Jordan, as a proxy for potential CL distribution, to (i) identify areas of highest CL risk and (ii) estimate the human population at risk of CL infection in regions of highest vector suitability. This would help address some of the actions outlined within the WHO’s ‘*Framework for action on cutaneous leishmaniasis in the Eastern Mediterranean Region 2014–2018’* [32] namely, recommendations regarding identification of high risk areas and targeted surveillance to minimise the burden of CL in Jordan.

## Materials & Methods

### Ethical Statement

This study received ethical approval by the Clinical Research and Ethical Review Board at the Royal Veterinary College (URN 2017 1673–3).

### Study area

The Hashemite Kingdom of Jordan is in the Middle East, approximately 100 km from the south-eastern region of the Mediterranean Sea, and shares borders with Israel, the State of Palestine, the Syrian Arab Republic, Iraq and Saudi Arabia. The country covers approximately 98,200 km^2^ of land with a population of approximately 9.5 million in 2016 (not including the refugee population), subject to a 3.2% annual population growth [58].

**Fig 1:**
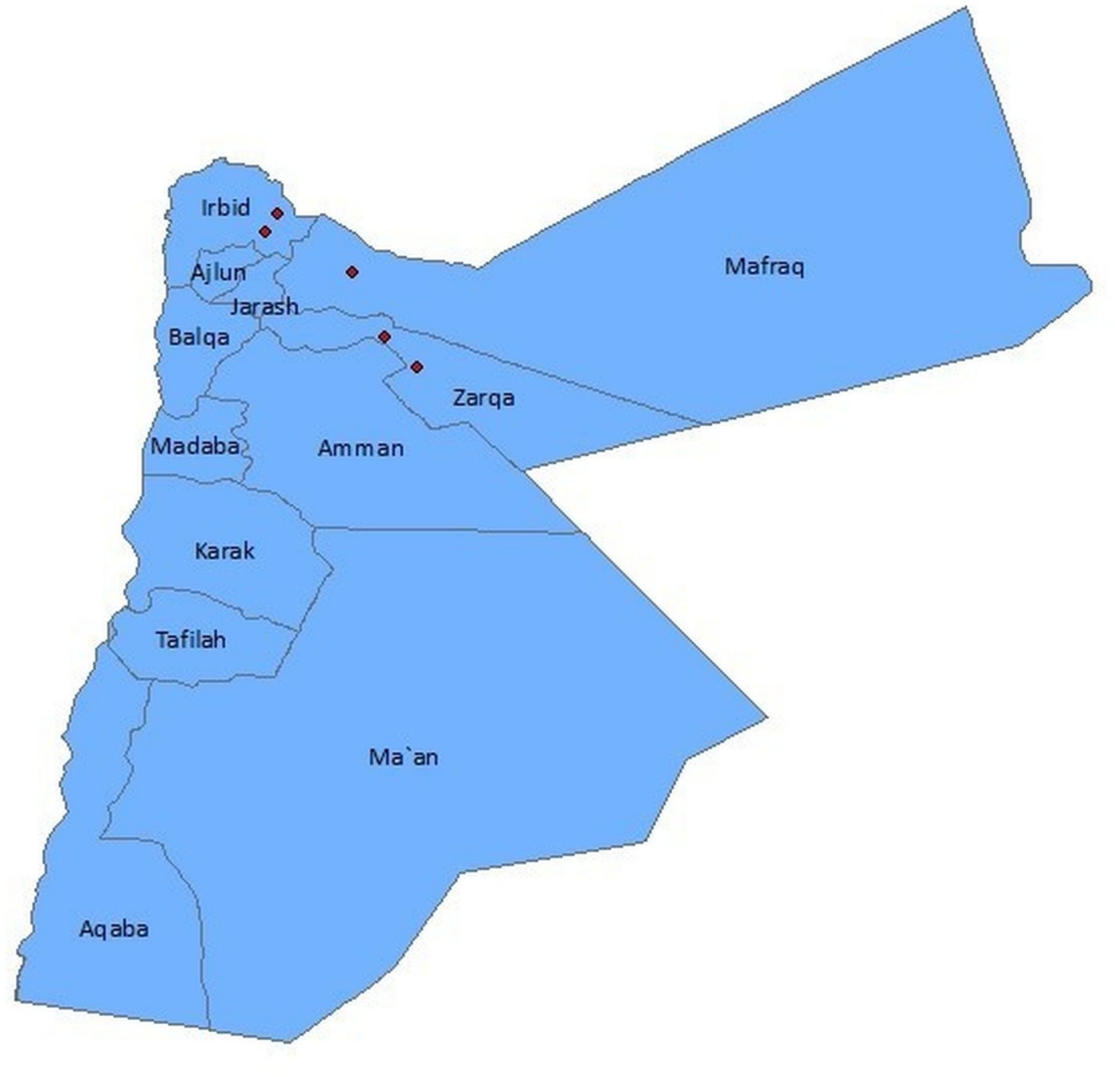
Map of Jordan’s Governorates and locations of Syrian refugee camps (red dots).

Administratively, Jordan is divided into twelve governorates (Fig 1) and can also be divided into four biogeographic zones—Mediterranean, Irano-Turania, Saharo-Arabian and Sudania—which all vary geologically and biologically [59]. Western Jordan, where the Jordan Valley is located, has a Mediterranean climate with hot dry summers and cool wet winters. It is the most fertile region, containing the Jordan River, and has thus witnessed the most intense agricultural development in the country. Conversely, between 75–80% of the country is classified as arid or semi-arid receiving less than 100 mm of rain annually [59]. The Mountain Heights Plateau extends across the entire length of the western part of the country, separating the Jordan Valley from the eastern desert plains and contains Jordan’s main population centres (Amman, Zarqa, Irbid and Karak). This area receives the highest rainfall and is therefore the most richly vegetated. Finally, the Badia region comprises approximately 75% of the country and is an area of desert and desert steppe, part of which is known as the North Arab Desert. Climate fluctuates widely with daytime summer temperatures exceeding 40°C, while winter nights can be very cold, dry and windy. Rainfall is minimal, averaging less than 50 mm annually [59].

### Multicriteria decision analysis

#### The MCDA method

The steps involved in spatial MCDA have been described in detail elsewhere [35,48]. Briefly, this method consists of the identification of georeferenced risk factors associated with a specific outcome using knowledge (existing or hypothetical) of their relationship with the specified outcome (in this study, the outcome was the predicted presence of *P*. *papatasi* in Jordan). Spatial risk factor data layers are initially processed before inclusion in the model. All layers are standardized to have equal pixel size and resolution, and varying scales of the different predictors are standardized using fuzzy membership functions to achieve a common continuous scale. Fuzzy functions measure the degree of data cell membership within a predictor layer through predefined control points based on the association (known or hypothetical) between the predictor variable and the outcome. These associations define the shape (J-shaped, sigmoidal or linear) and direction (increasing, decreasing, or symmetric) [48] of the hypothesized relationship between predictor and outcome. The resulting standardized layers are scaled across the entire range of values and the use of fuzzy functions operates on the basis that membership or non-membership of a location is gradual or can be partial [53,60].

If the model is to include only two equally important risk factors these can be combined using the Boolean operators “AND” or “OR”. On the other hand, if the model is to include two or more risk factors that differ in importance, each risk factor can be assigned a weight using pairwise comparisons. This will break down information whereby only two criteria are considered at a time, and ultimately result in stronger risk factors influencing the modelled outcome to a larger degree [61]. The final modelled outcome map is a result of a suitability estimation calculated for each spatial unit (i.e. raster cell) using the following equation where S = suitability, *w_i_* = weight of factor *i*, and *x_i_* = criterion score of factor *i*:

$$S=\sum w_{i}x_{i}$$

The suitability of each spatial unit is expressed on a continuous scale with lower values showing low suitability and high values representing areas of higher suitability.

#### Identification of predictor variables associated with *Phlebotomous papatasi* distribution

A literature search in Pubmed using the term ‘*Phlebotomus papatasi*’ identified 718 titles. These were screened at title, abstract and full text level and only publications in English that described the distribution and ecological conditions under which *P*. *papatasi* occurred and thrived were considered. Screening was carried out by a single reviewer (the first author) based on their subjective perception regarding the relevance of publications relating to bio-ecological information on *P*. *papatasi* occurrence. Title and abstract-level screening rejected studies that did not provide any information on spatial ecological determinants for *P*. *papatasi* occurrence and distribution. Rejected publications focused mostly on molecular mechanisms of *Leishmania spp*. infection, anatomical and morphological descriptions of *P*. *papatasi*, and the use of insecticides for vector control. Publications accepted after abstract screening (n = 135) were used to identify potential predictor variables while those accepted after full text screening (n = 52) were used to help define predictor range values for the model (median, minimum, maximum). These 52 publications included laboratory-based studies or field-based studies carried out in countries within North Africa and the Middle East (i.e. similar to Jordan).

The 135 publications used to identify potential predictor variables identified only seven spatial factors found to be associated with the occurrence and survival of *P*. *papatasi*, and taken forward to the MCDA model including temperature, humidity, precipitation, vegetation, wind speed, presence of human households and presence of the fat sand rat (*Psammomys obesus)*. Table 1 lists these seven predictor variables and the relationship between each and the occurrence of *P*. *papatasi*.

**Table 1:** List of model predictor variables. A description of variables identified by previous studies and publication sources as influencing the distribution of *Phlebotomus papatasi* and their relationship with its occurrence.

Variable	Variable name	Relationship with occurrence of *Phlebotomus papatasi*
Mean monthly temperature (C°)	Temperature	Sandflies are ectotherms and thus, temperature strongly influences developmental rates, survival and longevity [82, 83].
Fat sand rat distribution	*Psammomys obesus*	The fat sand rat (*Psammomys obesus)* is an important reservoir host for *Leishmania major*. *P*. *obesus* colonies and their burrows provide a source of food and refuge from extreme temperatures and desiccation [9,13,14,97]
Mean annual precipitation (mm)	Precipitation	Rainfall is associated with *P*. *papatasi* occurrence as it facilitates plant growth, which provides plant-based sugars for adult sandflies as well as food and shelter for rodents that also optimize *P*. *papatasi* survival [46].
Mean monthly relative humidity (%)	Relative humidity	*P*. *papatasi* require humid environments, especially larvae and pupae that lack protective mechanisms against water loss and are extremely susceptible to desiccation. Therefore, *P*. *papatasi* seek refuge and lay eggs in cracks within rocks and houses, and rodent burrows. Furthermore, both larval and adult stages’ survival increases with higher relative humidity levels [82].
Monthly vegetation	Normalized difference vegetation index (NDVI)	Vegetation provides plant-based sugars for adult sandflies as well as food and shelter for rodents that also influence *P*. *papatasi* occurrence [46]. It additionally is an indicator or soil moisture, which optimizes larval survival within burrows.
Wind speed (m/s)	Wind	Wind restricts sandfly activity and they are found more abundantly in areas sheltered from wind or when wind velocity is reduced [12,79,98,99].
Human settlement	Human settlement	*P*. *papatasi* benefits from human settlement as it provides food sources and shelter. Studies have found positive relationships between *Phlebotomus papatasi* occurrence and the expansion of human settlements [84,100]. Furthermore, *P*. *papatasi* is considered to be highly endophilic [45,79,81,100]; they are readily found in human domestic environments.

#### Data sourcing and manipulation

Georeferenced maps were sourced for each predictor variable where possible, and a proxy sought if there was no available georeferenced map for the original variable (Table 2). All maps were standardized to a common coordinate reference system (World Geodetic System 1984 (WGS_84)) and resampled to a resolution of 0.00833 by 0.00833 decimal degrees. The resampling technique used for the categorical human settlement data was the nearest neighbour algorithm while a bilinear algorithm was used for all continuous data (the remaining variables). Jordan’s administrative areas were obtained from the Global Administrative Areas website [62], and the country level administrative area was used to clip Jordan from global maps or region tiles. ArcMap 10.4.1 [63] was used for all spatial data manipulations.

**Table 2:** Georeferenced data sources and manipulations for predictor variables.

Variable	Georeferenced map source	Data manipulations
Temperature (C°)	WorldClim version 2 at a 30 arc second spatial resolution (approximately 1 km^2^) [64]	Clip Jordan country administrative area from global map; standardize resolution.
*Psammomys obesus*	No map available so proxy was generated for inclusion in model	MCDA for suitability distribution of the fat sand rat’s main food source *Anabasis articulata*.
Precipitation (mm)	WorldClim version 2 at a 30 arc second spatial resolution (approximately 1 km^2^) [64]	Clip Jordan country administrative area from global map; calculate annual precipitation by the total sum of all months; standardize resolution
Relative humidity (9am) (%)	CliMond climate dataset at a 10 arc minute spatial resolution [65]	Clip Jordan country administrative area from global map; standardize resolution
NDVI	Integrated Climate Data Centre (ICDC), Hamburg University [101].	Clip Jordan country administrative area from region tile; calculate average NDVI between 2000–2016 for each month
Wind (m/s)	WorldClim version 2 at a 30 arc second spatial resolution (approximately 1 km^2^) [64]	Clip Jordan country administrative area from global map; standardize resolution
Human settlement	European Commission’s Global Human Settlement Layer (GHSL) at a 1 km^2^ spatial resolution [102]	Clip Jordan country administrative area from global map; standardize resolution

WorldClim and CliMond use historical climate data and mathematical modelling techniques to create georeferenced raster maps for global mean monthly temperature, mean annual precipitation, mean monthly relative humidity and mean monthly wind speed [64,65]. In order to assess the degree of validity of modelled and actual climate data, simple linear regression analysis was carried out in R [66] between the modelled climate data (WorldClim and CliMond) and meteorological station data to measure the level of agreement between the two. National weather station data were requested from the Jordan Meteorological Department, and values underlying the 20 (24 for precipitation) weather station coordinates were extracted from temperature, relative humidity, precipitation and wind speed raster data maps (Table 3).

**Table 3:** Climate data validation variables. Description of climate variables, number of weather stations, and years for which data were acquired from the Jordan Meteorological Department.

Variable	Years
Mean monthly temperature	2010, 2012, 2014, 2016
Annual precipitation	2005–2011
Mean monthly relative humidity	2010, 2012, 2014, 2016
Mean monthly wind speed	2004, 2006, 2008, 2010, 2012, 2014, 2015

*P*. *obesus* distribution: As there was no available georeferenced map for the distribution of *P*. *obesus* in Jordan a proxy was sought. *P*. *obesus* is a specialist feeder and its occurrence is determined by the distribution of succulent plant species such as those of the *Chenopodiaceae* family [67,68]. The distribution of *Anabasis articulata* was thought to be a good proxy for *P*. *obesus* occurrence as it is an important component of *P*. *obesus’* diet in the Middle East [68–70]. Furthermore, *A*. *articulata* also forms part of *P*. *papatasi’s* diet [12]. To generate this map, a separate MCDA for *A*. *articulata* suitability distribution was performed. Soil properties describing soil texture and chemical composition (soil pH, percentage sand, clay and silt), and normalised difference vegetation index (NDVI) were identified from the published scientific literature as potential predictor variables for *A*. *articulata* occurrence [71].

Soil profile control points were defined using data from a study that measured soil properties at succulent vegetation growth sites, including *A*. *articulata*, in Egypt between 2004 and 2007 [71]. Georeferenced data were sourced for soil profiles in Jordan from the International Soil Reference and Information Centre (ISRIC) including soil pH, percentage sand, percentage clay and percentage silt [72]. El-Ghani *et al*. ([71]) carried out soil sampling between 0–50 cm deep thus, the ISRIC dataset was manipulated to create a new variable showing average values between soil layers D1 (0–19 cm) and D2 (20–39 cm). This new variable was joined to the original dataset and individual raster maps for each soil property (soil pH, percentage sand, percentage clay and percentage silt) were created using the updated dataset.

An NDVI map with average values between April and September (see Section 3.2 for reasoning behind this selection of months) was also included in the MCDA. All maps were standardised to a common scale by applying fuzzy membership functions (see Section 2.2.4): a symmetric sigmoidal function was applied to each soil property using minimum, median and maximum values identified from the literature, as control points, and a monotonically increasing sigmoidal function was applied for NDVI values between 0.2 and 0.3 (corresponds to shrubs and therefore thought to most likely to represent *A*. *articulate)*.

#### Creation of fuzzy sets

All maps were transformed and standardized to a common scale of 0–1 using fuzzy membership functions built on fuzzy logic [48,73] which assumes that membership of elements is not binary but can be gradual and/or partial (Table 4). In this way, uncertainty regarding the association between predictor variables and outcome is modelled using a fuzzy membership function to describe the shape of the hypothesized relationship between individual predictors and the outcome.

**Table 4:** Predictor variables’ fuzzy membership functions. Inclusion of the associated rationale used to convert the predictor variables into fuzzy sets for inclusion in the multicriteria decision analysis model.

Predictor variable	Rationale
Temperature	Laboratory experiments that determined *Phlebotomus papatasi’s* thermal lower and upper limits suggested them to be 10°C when cold paralysis appeared, and 39.5°C when all individuals died [82]. However, at temperatures below 15°C no larvae reached pupal stage [83]. Field data from Iran, Egypt and Libya reported sandfly abundance peaks at temperatures between 25°C and 30°C [12,103–105]. Therefore, a symmetrical sigmoidal relationship was applied using the control points 15°C, 25°C, 30°C, 39.5°C.
Precipitation	Studies showed that there was lower probability of *Phlebotomus papatasi* occurrence when annual precipitation was lower than 50 mm [38, 44]. Abundance was recorded to be higher in areas where precipitation ranged between 100 mm to 600 mm with a maximum recording of 947 mm [44,46]. Therefore, a symmetrical sigmoidal relationship was applied using the control points 50 mm, 100 mm, 600 mm, 947 mm.
NDVI	NDVI values range from -1 to +1; clouds and snow are characterized by negative values while soils, rock and vegetation are positive. Barren areas of rock, sand or soils exhibit very low values (0.1 to 0.2), shrub and grassland represent moderate values (0.2 to 0.3) while temperate and tropical rainforests have high values (0.6 to 0.8) [106]. Therefore, in this study a linear monotonically increasing relationship between 0.2 and 0.6 was applied. Higher values would not be expected in arid or semiarid regions like Jordan, with the higher end values most likely corresponding to agricultural land.
Relative humidity	Adult *Phlebotomus papatasi* are able to survive at all relative humidity values 0–100%; however, the higher the relative humidity the higher the longevity [82]. Therefore, a monotonically increasing linear relationship ranging between 0% and 100% was applied.
*Psammomys obesus*	*Psammomys obesus* provides shelter and food sources for *Phlebotomus papatasi*, which is found around *Psammomys obesus’* burrows [9,13,14,97]. *Anabasis articulata* occurrence was used as a proxy for *Psammomys obesus’* distribution[67,68], and a monotonically increasing linear relationship was applied using the MCDA output for the suitability distribution of *Anabasis articulata*. Control points were set at 0.1 and 1; the upper limit corresponding to the maximum MCDA value.
Wind	Studies found sandfly activity to be highest when no wind was present, reduced when velocity was above 2 m/s and ceased when above 4 m/s [12,107,108]. Therefore, a monotonically decreasing sigmoidal relationship was applied using the control points 2 m/s and 4 m/s
Human settlement	GHSL data was categorical; urban centre (3), urban cluster (2), and rural area (1). The positive relationship between *Phlebotomus papatasi* occurrence and human settlements [84,100] led to applying a monotonically increasing linear relationship from rural areas (1) to urban centres (2).

#### Predictor weights

Weights were calculated using Saaty’s AHP [47] for both *P*. *obesus* and *P*. *Papatasi*. After standardisation to a common scale (0–1) predictor weights were determined using the pairwise comparison matrix of the AHP [61] (Table 5) whereby (i) predictor weights are derived by taking the principal eigenvector of a square reciprocal matrix of pairwise comparisons between the criteria, and (ii) a consistency ratio (CR) is calculated [61]. Pairwise comparisons were made using a 9-point scale (Table 5) comparing the subjective relative importance of the variable in the first column to the rest of the predictor variables. If the rating for the *yX* cell was 5 it would mean that *y* is strongly more important than *X* in determining the suitability of the outcome (i.e. the occurrence of *P*. *papatasi)*. The combined total of all weights summed to one. Saaty (61) proved that for a consistent reciprocal matrix, the largest Eigenvalue (*λ_max_*) needed to be equal to the size of the comparison matrix (n), or *λ_max_* = *n*. Based on this principle, he went on to propose a method to calculate a consistency ratio that included a consistency index (CI) and random consistency index (RI). The CI is a measure of consistency and is defined as:

$$CI=\frac{\lambda_{max}-n}{n-1}$$

The CI is then compared to the RI, which is a randomly generated reciprocal matrix that uses different scales in a similar way to bootstrapping. Finally, the consistency ratio (CR) is defined as

$$CR=\frac{CI}{RI}$$

The CR measures the consistency of judgements in relation to large samples of random judgements [61]. If CR>0.1 the judgements are close to randomness and the AHP should be re-evaluated.

**Table 5:** Rating scale used for pairwise comparisons between predictor variables.

**Pairwise comparison 9-point continuous rating scale**								
1/9	1/7	1/5	1/3	1	3	5	7	9
Extremely	Very strongly	Strongly	Moderately	Equally	Moderately	Strongly	Very Strongly	Extremely
**LESS IMPORTANT**					**MORE IMPORTANT**			
		
**Predictor variables**					**X**	**Y**		**Z**
**x**								
**y**					yX			
**z**					zX	zY		

Owing to the subjective nature of the pairwise comparison method, an expert elicitation was used initially to minimize bias. Experts within the field of *P*. *papatasi* ecology in the Middle East were identified from the previously conducted literature review to identify *P*. *papatasi* predictor variables, and through international conferences such as WorldLeish2017 (Toledo). Six experts were contacted via email and provided with a brief explanation of the objectives and methods of the study along with an uncompleted pairwise comparison table (Table 6). In addition to rating the relative importance between predictor variables in relation to the specified outcome using the scale presented in Table 5, a confidence score ranging between 1 (not confident) to 5 (most confident) was to be included by the experts for each rating within the matrix to quantify the assessor’s confidence in their score. The final pairwise comparison matrix from which predictor weightings were calculated, used the weighted geometric mean of each of the expert’s subjective opinion using the confidence score as a weight:

$$\bar{x}=exp\;\left(\frac{\sum_{i=1}^{n}w_{i}\;\ln x_{i}}{\sum_{i=1}^{n}w_{i}}\right)$$

where *x_i_* = pairwise comparison rating

*w_i_* = confidence score

However, as the expert elicitation resulted in a CR>1 for all experts and were therefore considered to be little better than random, it was decided that pairwise comparisons for both *P*. *obesus* and *P*. *Papatasi* should instead be made by the first author, who had completed the literature review of factors associated with vector occurrence and survival

**Table 6:** Pairwise comparison matrix of the analytical hierarchy process (AHP) for the predictors associated with the occurrence of *Phlebotomus papatasi* in Jordan. Based on the first author’s subjective judgment constructed from the literature review in Section 2.2.2.

	Temp	Relative humidity	Precip	NDVI	*Psammomys obesus*	Wind	H. Sett.	Weight
**Temperature**	1							0.3590
**Relative humidity**	1/7	1						0.0332
**Precipitation**	1/5	3	1					0.0615
**NDVI**	1/5	3	1	1				0.1210
***Psammomys obesus***	1/3	5	3	3	1			0.1210
**Wind**	1/3	3	3	3	3	1		0.1774
**Human settlement**	1/3	5	3	3	3	1	1	0.1864

* Consistency ratio = 0.05; Temp = temperature; Precip = precipitation; H. Sett = human settlement; NDVI = normalized difference vegetation index

#### MCDA model

Fuzzy sets and predictor weightings were obtained using the Terrset Geospatial Monitoring and Modelling software [74]. Once all six predictor variables were (a) identified; (b) sourced and georeferenced; (c) standardized to a common size, resolution and scale using fuzzy membership functions; and (d) weighted using the AHP, the MCDA model was run to produce a final map illustrating the predicted habitat suitability for *P*. *papatasi* in Jordan. Suitability values ranged between 0 and 1 with higher values indicating higher suitability. Individual suitability maps were produced for the months between April and September (see Section 3.2 for the rationale behind selection of these months).

#### Sensitivity analysis

A sensitivity analysis was carried out to explore how changes in weights and membership functions for each predictor variable would affect the final suitability estimate. To investigate the effect of membership function, the model was run assuming a linear association between all predictor variables and the occurrence of *P*. *papatasi*. Likewise, to investigate the effect of a change in weights, the model was run with equal weights applied to all predictor variables. Final suitability estimates were extracted from 100 random points from the original and new maps, and mean changes between these values were compared using Student’s t-tests (histograms and QQ plots showed all variables were normally distributed) using IBM SPSS Statistics 23.

### Human population at risk of *P*. *papatasi* exposure

Binary maps depicting high versus low *P*. *papatasi* suitability were created using different cut-off values (0.5, 0.6, 0.7, 0.8 and 0.9) for each month between April and September, before taking an average of the six months for each cut-off value. The population at risk (PAR) of *P*. *papatasi* exposure was defined as the total population residing in areas identified as suitable (above 0.5, 0.6, 0.7, 0.8 or 0.9) for *P*. *papatasi* occurrence. To calculate this, two different gridded population maps were used: the European Commission’s Global Human Settlement (GHS) population grid (http://ghsl.jrc.ec.europa.eu/ghs_pop.php), and the Gridded Population of the World (GPW) (http://sedac.ciesin.columbia.edu/data/set/gpw-v4-population-count-adjusted-to-2015-unwpp-country-totals). Different population datasets were used to account for the uncertainty associated with different gridded human population datasets [75].

## Results

### *Psammomys obesus* distribution

**Fig 2:**
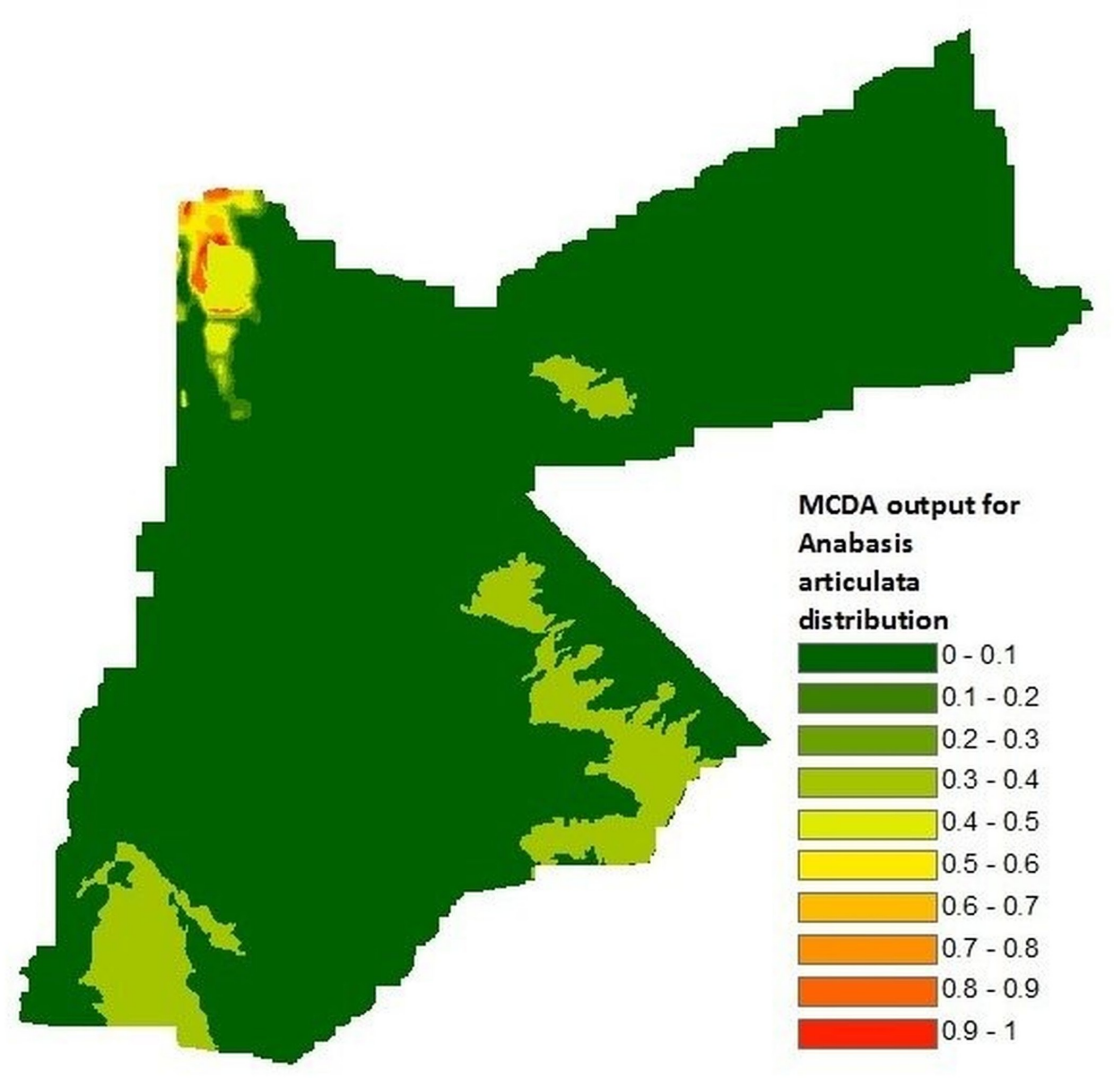
Multicriteria decision analysis output for the suitability distribution of *Anabasis articulata*. This was used as a proxy for the distribution of *Psammomys obesus*; a predictor variable for the occurrence of *Phlebotomus papatasi*.

The AHP assigned a weight of 0.5556 for vegetation and 0.1111 for each of the soil properties (Table 7). The resulting MCDA produced the estimated distribution of *A*. *articulata* displayed in Fig 2. Suitability values ranged between 0–1 with lower values (green) indicating lower suitability, and conversely higher values (red) indicating higher suitability for occurrence of *A*. *articulata*. High suitability areas were concentrated in the north-west region of the country.

**Table 7:** Pairwise comparison matrix of the analytical hierarchy process (AHP) for the predictors associated with the occurrence of *Psammomys obesus* in Jordan. Based on the first author’s subjective judgement constructed from published literature.

	NDVI	pH	Clay (%)	Sand (%)	Silt (%)	Weight
**NDVI**	1					0.5556
**pH**	1/5	1				0.1111
**Clay (%)**	1/5	1	1			0.1111
**Sand (%)**	1/5	1	1	1		0.1111
**Silt (%)**	1/5	1	1	1	1	0.1111

* CR = 0.00

### Climate data validation

**Fig 3:**
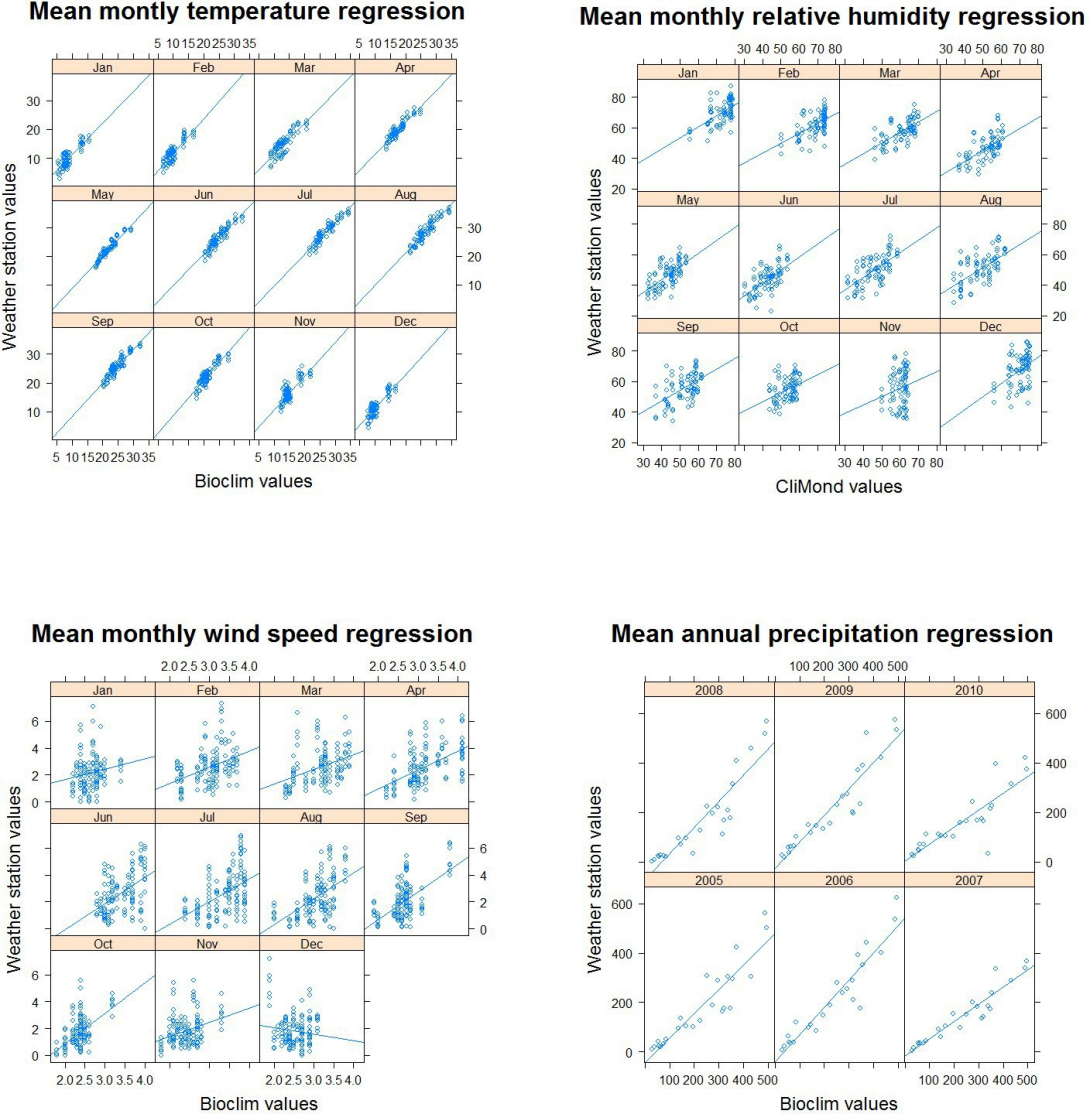
Data validation regression plots. Results from simple linear regression analysis between weather station climate data recordings and modelled climate data using historical recordings for (A) mean monthly temperature, (B) mean monthly relative humidity, (C) mean monthly wind speed, and (D) mean annual precipitation.

Linear regression between weather station data and modelled climate data gave different results for different variables. Mean monthly temperature and mean annual precipitation showed the highest levels of association and agreement (Fig 3A–3D) as demonstrated by their high R^2^ values (Table 8). For mean monthly relative humidity and mean monthly wind speed, the association was statistically significant for most months (Fig 3B and 3C) but R^2^ values fluctuated and were lower compared to mean monthly temperature and mean annual precipitation (Table 8). The results also showed that data on warmer months were more reliable, indicated by higher R^2^ values (Table 8). The suitability model was therefore run only for the months between April and September. This coincided with the timeframe of most interest from an epidemiological perspective, as this is when *P*. *papatasi* is most abundant [76–78].

**Table 8:** Climate data validation results. R^2^ values derived from the simple linear regression analysis between weather station climate data recordings and modelled climate data using historical recordings. Overall, R^2^ values were higher for the warmer months of the year (April-September).

Month/Year	Mean monthly relative humidity	Mean monthly wind speed	Mean monthly temperature	Mean annual precipitation
January	0.31*	0.02°	0.7*	
February	0.3*	0.15*	0.73*	
March	0.4*	0.14*	0.71*	
April	0.35*	0.3*	0.86*	
May	0.4*	0.3*	0.9*	
June	0.38*	0.24*	0.84*	
July	0.42*	0.22*	0.88*	
August	0.38*	0.28*	0.85*	
September	0.27*	0.31*	0.86*	
October	0.13*	0.31*	0.79*	
November	0.02	0.1*	0.67*	
December	0.17*	0.01	0.72*	
2005				0.83*
2006				0.88*
2007				0.89*
2008				0.83*
2009				0.87*
2010				0.72*

* P-value < 0.001

° P-value < 0.05

### Predictor variables

**Fig 4:**
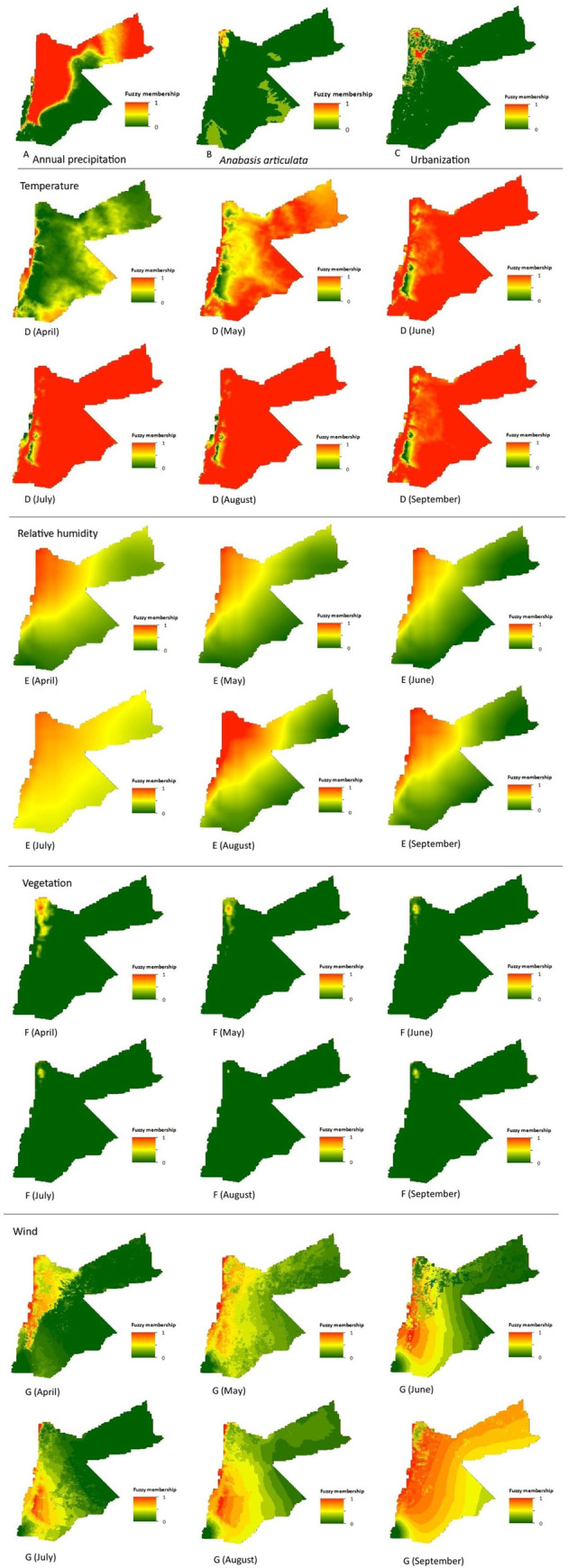
Fuzzy membership maps for each predictor variable. (A) Annual precipitation, (B) *Anabasis articulata*, (C) human settlement, (D) temperature, (E) relative humidity, (F) vegetation and (G) wind.

Fuzzy membership functions were applied to each predictor variable. Moreover, for those predictor variables for which monthly data were available, fuzzy membership functions were applied to each month between April and September. Values ranged from 0 (green) to 1 (red) indicating the degree of membership of suitability of each raster cell for *P*. *papatasi* occurrence (Fig 4). Membership for *P*. *papatasi* occurrence suitability in terms of annual precipitation (Fig 4A), was widespread but low in the south and south-eastern parts of the country, where rainfall is scarce. For *A*. *articulata* (proxy for *P*. *obesus*) (Fig 4B) membership was higher in the north-western tip of the country. Finally, membership regarding human settlement (Fig 4C) was also concentrated in the north-western parts of the country in line with the location of the largest most populated cities such as Amman, Irbid and Zarqa. Monthly fluctuations concerning cell membership for *P*. *papatasi* suitability were observed for the remaining environmental predictor variables. Larger areas of the country became suitable in terms of temperature as summer progressed (Fig 4D). Cell membership associated with relative humidity was higher in the north-west but became more widespread throughout the country during July (Fig 4E) while cell membership linked to vegetation was focussed mostly in the north-western tip of the country and decreased over the summer months (Fig 4F). Cell membership associated with wind showed a peak along the Jordan Valley at the western border of the country with higher widespread membership in September due to reduced wind speed for this month (Fig 4G).

### Weights

The completed pairwise comparison matrix for the AHP is detailed in Table 6. The highest weighted predictor variable was temperature followed by human settlement and wind speed, while relative humidity was the lowest weighted predictor variable. The consistency ratio was 0.05.

### Suitability distribution of *P*. *papatasi* in Jordan

**Fig 5:**
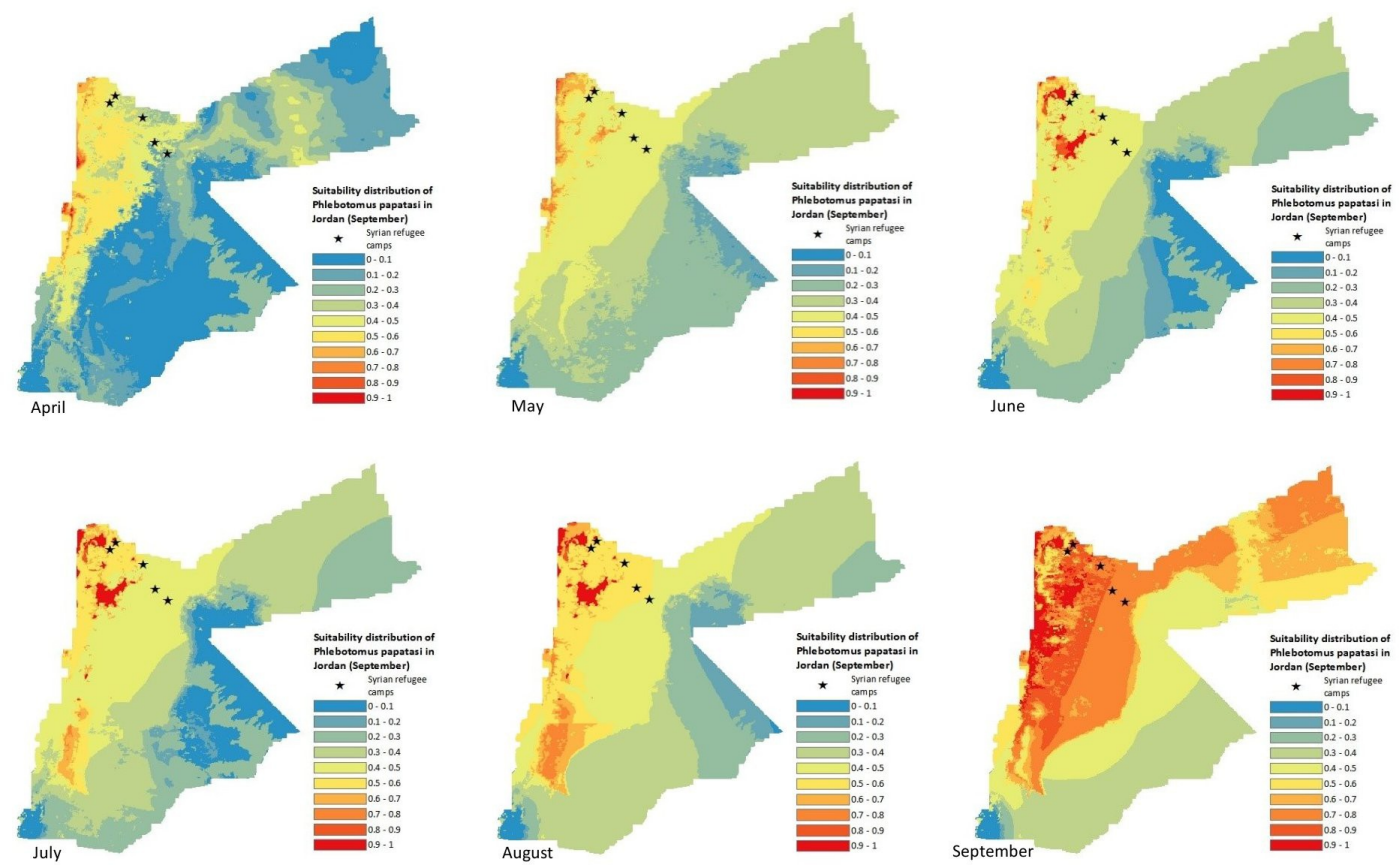
MCDA outputs for the predicted suitability distribution of *Phlebotomus papatasi* in Jordan from April to September. * show the location of Syrian refugee camps.

Suitability for the occurrence of *P*. *papatasi* in Jordan for the different months between April and September is presented in Fig 5 using a continuous 0 to 1 scale. Blue areas were areas of very low or low suitability; yellow and light orange areas were moderately suitable, while dark orange and red areas were considered highly suitable for *P*. *papatasi* occurrence. In general, suitability was higher in the north-western areas and along the Jordan Valley to the west, while suitability was generally lower in the eastern and southern regions of the country. Suitability increased with the progression of summer and September showed the highest and most widespread suitability. Syrian refugee camps (depicted as black stars) were in areas of low to moderate *P*. *papatasi* suitability except for September. However, those located more towards the northwest were in very close proximity to areas displaying high suitability.

**Fig 6:**
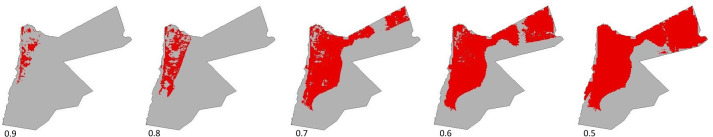
Geographic areas suitable for *Phlebotomus papatasi* occurrence using suitability cut-offs of 0.9 to 0.5.

The extent of geographic areas suitable for *P*. *papatasi* occurrence varied according to suitability cut-offs; areas defined by a higher suitability cut-off were smaller and more dispersed (Fig 6A and 6B) compare to areas defined by a lower or moderate suitability cut-off (Fig 6C–6E), which were more homogenous and extensive.

### Sensitivity analysis

In general, the change in fuzzy membership functions caused a larger mean change than changing the weights (Table 9). Equal weights of 0.143 for each of the seven predictor variables produced an overall mean change of 0.06 ± 0.13 units, while applying a linear relationship for all predictor variables resulted in a mean change of 0.09 ± 0.16 units.

**Table 9:** Sensitivity analysis results. Mean change in values (n = 96) between original multicriteria decision analysis outputs compared to new maps with either equal weights for all predictor variables, or assuming linear membership functions for all predictor variables.

Month	Mean change for equal weights	Mean change for linear membership function	P-value
April	0 ± 0.11	0.07 ± 0.12	< 0.01
May	0.05 ± 0.06	0.06 ± 0.1	> 0.05
June	0.07 ± 0.14	0 ± 0.12	< 0.01
July	0.02 ± 0.09	0.12 ± 0.15	< 0.01
August	0.05 ± 0.17	0.12 ± 0.2	< 0.01
September	0.18 ± 0.11	0.18 ± 0.18	> 0.05
Overall	0.06 ± 0.13	0.09 ± 0.16	< 0.01

### Population at risk (PAR)

**Fig 7:**
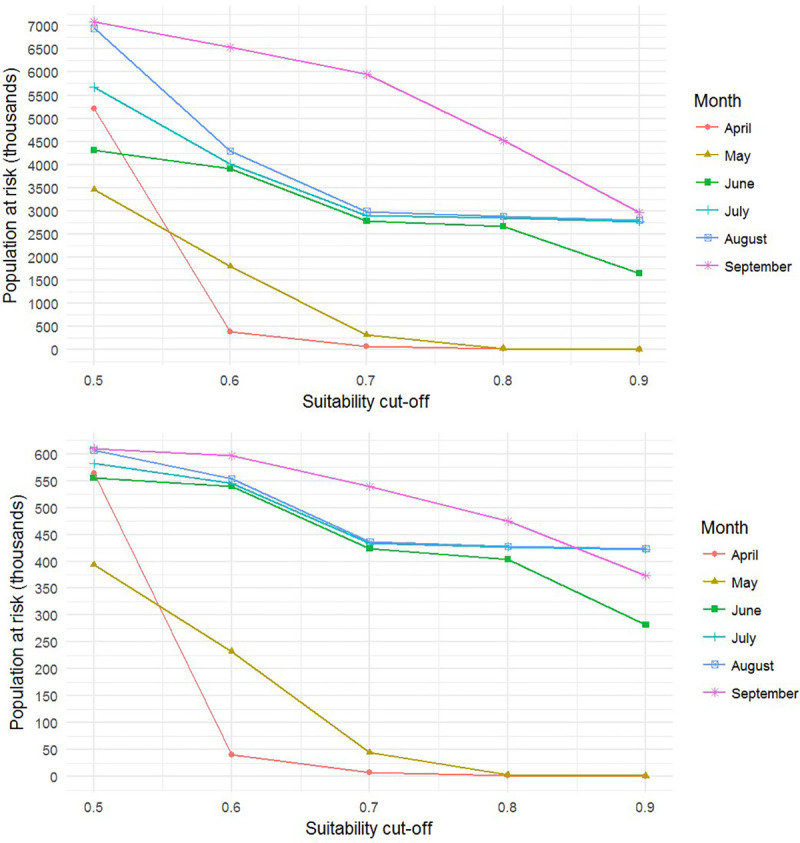
Population at risk of *Phlebotomus papatasi* exposure between April and September using different suitability cut-off points from moderate (0.5) to very high (0.9) suitability calculated using (top) the Global Population of the World (GPW) grid and (bottom) the European Commission’s Global Human Settlement (GHS) population grid.

The use of the two population grids (GHS and GPW) to calculate PAR for different months and at different suitability cut-offs, gave a similar pattern but at sizeably different scales (Fig 7). PAR was positively associated with the progression of summer; the number of people at risk of *P*. *papatasi* exposure was lowest in April and increased throughout the summer months with similar values during June, July and August, and finally September when PAR was highest. The GPW population grid presented a total national population of 7,536,579, while the GHS population grid presented a total national population of 4,600,878; a 39% difference. This translated into the GPW-based PAR calculation being of a substantially larger order of magnitude compared to GHS (millions versus hundreds of thousands; Fig 7).

However, the change in PAR in response to different suitability cut-offs was not proportional to the change observed for geographic range at risk. There was a large reduction in geographical area at risk ranging from 57% of the country at moderate (0.5) suitability, to 3% of the country at very high suitability (0.9). However, the change in PAR was more modest; according to GHS and GWP population grids 13% and 100% of the total Jordanian population were at moderate (0.5) risk, while 10% and 90% were at very high risk (0.9) respectively (Table 10).

**Table 10:** Population at risk results. Quantification of (i) areas suitable for *Phlebotomus papatasi* occurrence in relation to the whole country; (ii) the population at risk of cutaneous leishmaniasis using different population grids; and (iii) the population at risk in relation to the total population, using different suitability cut-off values.

Suitability cut-off	% of Jordan within suitability range	PAR (GHS)	% of total Jordan population at risk (GHS)	PAR (GPW)	% of total Jordan population at risk (GPW)
0.5	57.44	610,100	13.26	7,535,141	99.98
0.6	46.77	598,801	13.01	7,506,769	99.60
0.7	34.92	550,408	11.96	7,422,532	98.49
0.8	10.35	480,652	10.45	7,217,799	95.77
0.9	3.42	443,269	9.63	6,749,171	89.55

## Discussion

This is the first study, to the author’s knowledge, to explore the distribution of the main CL vector *P*. *papatasi* in Jordan. It adds to the limited number of studies that have investigated CL vector distribution in the Middle East using spatial analysis tools, none of which used MCDA [41,44–46]. In addition, this study separates itself from past studies investigating CL vectors by including a set of carefully selected variables using evidence-based biological rationale rather than being restricted to variables for which data are available. Other predictive studies on CL vector distribution have mostly included readily available variables such as the WorldClim bioclimatic variables [41–43,45,46], which are easily accessible and provide a range of resolutions, the highest being 1 km^2^. Despite the quality of WorldClim data, of the available 19 bioclimatic variables, 11 are temperature-related and the remaining 8 are precipitation-related. Therefore, the diversity of environmental variables included in previous predictive models is limited.

In addition to the inclusion of a comprehensive range of biologically-backed predictor variables, this study calculated the population at risk of CL, which has never been undertaken in Jordan. This is a key metric considering the current demographic changes that are taking place in Jordan, especially with conflict-related displacement in neighbouring countries.

### Habitat suitability for *P*. *papatasi* occurrence

The results showed higher habitat suitability for CL vector occurrence in the north-western areas and along the Jordan Valley to the west of the country. *P*. *papatasi* have previously been found in neighbouring adjacent regions along the Dead Sea and the Jordan Valley in Israel and the State of Palestine [9,14,79–81], which reaffirms the suitability of these nearby areas to sustain *P*. *papatasi*. Additionally, regions most suitable for sustaining *P*. *papatasi* showed broad agreement with locations where CL cases have been reported historically [18,19,21–23].

The north-west region was suitable for *P*. *papatasi* occurrence as it presented optimal predictor variable values as shown in the fuzzy membership function maps (Fig 4). North-western areas had favourable temperatures, higher relative humidity, suitable locations for *P*. *obesus*, higher vegetation index, increased human settlement, higher annual precipitation, and lower wind speed. *P*. *papatasi’s* predicted distribution range increased throughout the summer months, which was expected due to the warmer temperatures enabling and optimising sandfly development [82,83]. The largest geographical range of high suitability areas was found in September due to the lower recorded wind speed, which led to more widespread favourable conditions.

High *P*. *papatasi* suitability areas coincided with the most populated regions in Jordan. This is of concern due to *P*. *papatasi*’s endophilic nature, being found indoors within domestic environments more frequently than other sandfly species [79,81,84]. This behavioural trait, in addition to facilitating CL transmission, could benefit *P*. *papatasi* survival by providing stable optimal conditions with minimal competition, as other sandfly species are not as endophilic. On the other hand, it could be advantageous from a vector control perspective as indoor residual spraying could be largely effective against an endophilic vector.

### Use of risk maps in cutaneous leishmaniasis mitigation strategies in Jordan

CL is an endemic and notifiable disease in Jordan. However, there is no national leishmaniasis control program and case detection is passive. The results from this study are valuable for addressing the actions outlined within the WHO East Mediterranean Region’s framework. Firstly, risk factors were synthesized, weighted and mapped. Furthermore, the integration of *P*. *papatasi* risk factors into a knowledge-driven habitat suitability model, identified risk areas for CL vector occurrence and thereby, CL transmission risk. The results revealed high human population densities in *P*. *papatasi* suitable areas. As such, implementing targeted control and surveillance measures in areas identified as having a very high CL vector suitability (<0.9 suitability) would include a substantial proportion (72% for GHS and 89% for GPW) of the population at risk while encompassing only 3.42% of the country’s total geographic area. Therefore, high impact public health interventions could be achieved within a reduced spatial target, thus maximizing the efficient use of resources.

Important drivers of emergence, re-emergence and/or expansion of vector-borne diseases include global travel, large scale human migrations and climate change. The former two are relevant in the context of epidemic/pandemic opportunities and instability-related migrations, while the latter may facilitate the extension of the spatio-temporal availability of favourable conditions for vector survival and reproduction. Jordan’s geographical location has resulted in it being a main destination for refugees fleeing violence in Syria. While endemic in both Jordan and Syria, CL has been reported considerably more frequently in Syria with a significant surge since the onset of political terror and the civil war [3,27,29,85], and the risk of a CL outbreak in Jordan may increase if potential CL-carriers are living in CL vector-suitable areas along with a large, possibly immuno-naïve, population.

The use of *P*. *papatasi* risk maps was a useful proxy for exploring risk of CL instead of directly using CL epidemiological data for several reasons. Firstly, CL reporting is carried out at governorate level so using epidemiological data would result in risk maps at a very coarse spatial resolution. Secondly, the possibly severe under-reporting of CL [26], which may be due to treatment-seeking socio-economical barriers or behaviours, might introduce bias to any map based on epidemiological data. Jordan’s MoH provides free healthcare to the Jordanian population and registered Syrian refugees. However, for those displaced Syrians who remain unregistered or do not possess any official documentation, access to adequate healthcare is challenging and dependent on a network of non-governmental organisations. Moreover, increased pressure on resources leading to shortage of medical staff and supplies has resulted in tensions between the Jordanian and Syrian populations, some of whom have resorted to seeking medical care in private clinics [86]. Because of inadequate and challenging access to healthcare, people might seek treatment only for severe or facial lesions as CL is generally known to be self-curing [32,87,88]. Therefore, the migration of Syrian displaced populations, including possible CL carriers, may drastically affect the national epidemiology but might not be captured as a consequence of the ongoing challenges to adequate healthcare access, under-reporting and passive surveillance [26,29,30,85,86,89]. The model outputs presented in this study could therefore, be used to highlight priority target areas to minimize CL burden. However, factors beyond the geographical distribution and determinants of disease such as financial cost, perceived risk, and existing policies and priorities in allocation of resources [35], need to be considered in public health decision-making.

### Risk maps and MCDA for surveillance and control of vector-borne diseases

Spatial risk models for CL, as with other vector borne diseases, are valuable in characterizing the entomological risk and thus, exposure to a disease vector. However, they are only one tool within a larger and more complex decision-making framework for the prevention and control of vector-borne diseases. Despite their limitations they are inexpensive, and in the absence of detailed field-based surveillance information, MCDA provides preliminary estimates of risk that, although imperfect, can be used for the initial stages of planning a control program or setting priorities [35]. In addition, this work can be easily updated with new information, which is an important advantage given the current changing global climate and population dynamics influenced by growth and migration.

Spatial MCDA has been used to set the level of risk for Rift Valley Fever (RVF) by incorporating environmental drivers, expert opinions and uncertainties to create continent-wide estimates of RVF activity [49]. Spatial MCDA has also been used to model habitat suitability for highly pathogenic avian influenza in south-east Asia [55] and low pathogenic avian influenza in Argentina [90]. Such work can be taken a step further by comparing two risk models, which was carried out by Sarkar *et al*. [50] to understand Chagas disease risk in Texas [51] where one model addressed vector distribution while a second model addressed parasite presence, allowing a more robust understanding of overall risk. MCDA could be applied to evaluate a set of vector control strategies to identify the best alternative among all possible options (e.g. pesticide spraying, promotion of protective behaviours such as using repellent or bed nets, or larvicide programmes). Rakotomanana *et al*. [50] used spatial MCDA to assess malaria risk in the highlands of Madagascar to identify the most effective use of resources for vector control. Decision-making criteria included elevation, population density, time since last indoor spraying, distance from rice fields and district level surface area of rice fields. The results from the spatial MCDA were then used to define priority zones to carry out indoor spraying [50].

### MCDA advantages and limitations

This study exemplifies how MCDA can be a powerful tool that can be applied to address complex decisions in data-scarce settings. MCDA is advantageous in that it can incorporate different and even conflicting views of several stakeholders and so criteria are chosen based on these values along with any potential constraints (e.g. reduction of disease-related morbidity/mortality, environmental impact, costs, duration of effectivity). This approach allows the integration of criteria that traditionally would not have been able to be considered simultaneously, and to rank alternative control strategies based on specified evaluation criteria.

However, there are limitations that need to be considered when interpreting these results. The most important consideration is that the quality of the model outputs will depend on the quality of the input data [33] and that the model might be subject to a degree of uncertainty that can be input-related linked to the source predictor data layers, and/or process-related resulting from data transformations and spatial interpolation algorithms [75]. The climatic data included in the model showed relatively good agreement with Jordan weather station data except for wind speed and relative humidity. This could have been due to the use of different measuring methodologies to capture wind speed as measurements depend greatly on the height at which they are measured, roughness of the ground and surrounding structures like buildings or trees [91]. Wind speed was a new variable only recently included within WorldClim V.2 (2017), so it might require time to optimize outputs and adjust errors. CliMond produced relative humidity maps at an arc 10’ spatial resolution, which might be too coarse to agree with weather station data. Population grids are highly variable and particularly inaccurate for low and middle income countries [75]. This is due to limited up-to-date demographic data as population censuses are usually carried out every ten years [75]. The marked difference between GHS and GPW population grids used in this study may be due to the use of different methodologies. An important difference includes the use of United Nations (UN)-adjusted population data in GPW, whereby the use of national statistic census data are adjusted to match official UN population estimates [92].

The second important consideration to keep in mind when interpreting MCDA outputs is the subjectivity involved in the methodology especially in (i) the selection of predictor variables; (ii) the definition of the relationship between predictor variable and outcome suitability using fuzzy membership functions; and finally (iii) the allocation of weights. An individual’s perspective on these subjects is expected to be influenced by personal experience and expertise although there are ways to address and ameliorate the possible bias and uncertainty that an individual may introduce. One option involves group discussions with experts and stakeholders to reach a group consensus. An alternative option involves the use of the scientific literature and allocating weights based on the number of times a variable has been reported along with its statistical significance [49,93] This study adopted a mixture between these two methods. Expert elicitation and group consensus was not possible due to the low CR of each of the returned questionnaires. The quantitative inclusion of some variables such as vegetation (NDVI), *P*. *obesus* distribution, wind speed or human settlement, are novel in *P*. *papatasi* ecology research which was reflected in the experts’ decreased confidence scores for these variables. For this reason the “*hit-based*” method applied by Stevens *et al*., 2013 [93] or Clements *et al*., 2006 [49] would not have been fully suitable either as important variables that influence *P*. *papatasi* occurrence are under-represented in the published literature. The restricted use of these variables in previous studies could be due to their limited availability or technical requirements associated with their use: WorldClim has only just recently included wind speed within its data repository; NDVI and human settlement maps require analysis of remote sensing satellite data; and a proxy was required and produced for *P*. *obesus* distribution. This highlights how data availability and resolution are important limitations for MCDA outputs. In addition, the range of predictors included in the model (e.g. vegetation (NDVI), *P*. *obesus* distribution, wind speed) means that to be effective, any expertise required would be from very diverse fields, therefore several panels of experts would be needed including botanists, zoologists, and entomologists, and therefore it was decided that these factors justified using a literature review across fields, and judgement by the first author.

Despite concerns associated with potential error due to the use of subjective risk factor weighting, this study showed that altering weights had a significantly lower effect on the final suitability estimate compared to altering the membership functions. These results are in line with findings by Stevens *et al*. (2013) [93] and Clements *et al*. (2006) [49], therefore emphasizing the importance of carefully considering and understanding the implications of the use of different membership functions.

A wide range of metrics are available to compare the veracity of model predictions with observations, whether based on a totally independent test data set, or on resampled observations within the training set, as in the case of cross-validation or bootstrapping. However, by their very nature these validation techniques assume the availability of both disease or vector presence and absence data; an inherent limitation in knowledge-driven modelling. However, model validation is inextricably linked with model uncertainty which can be either epistemic (e.g. measurement error, natural variability, model uncertainty) or stochastic (i.e. uncertainty due to inherent variability in the underlying biological processes [94,95]. Although uncertainty resulting from data errors can be substantial, of equal importance is the uncertainty resulting from missing predictors due to incomplete knowledge of the organism being modelled or because the spatial data for the predictor are unavailable [96]. This study has reduced this form of uncertainty and set itself apart from previous studies investigating CL vectors by including a set of carefully selected variables using evidence-based biological rationale rather than data availability. Other predictive studies on CL vector distribution have mostly included readily available variables such as the WorldClim bioclimatic variables [41–43,45,46], which are easily accessible and provide a range of resolutions; the highest being 1 km^2^. Despite the quality of WorldClim data, of the available 19 bioclimatic variables, 11 are temperature-related and the remaining 8 are precipitation-related. Therefore, the diversity of environmental variables included in previous predictive models is limited.

Estimates of uncertainty are generally only generated or considered at the end of the modelling process despite that, whenever possible, the concept should be incorporated at all stages [95]. In this respect MCDA may be considered to have an advantage over other modelling methods as the use of fuzzy logic and fuzzy membership functions inherently incorporates uncertainty about the relationships between predictors and outcome within the modelling process.

## Conclusions

Habitat suitability for the main CL vector *P*. *papatasi* in Jordan is heterogeneous over space and time. Regions in the north-west are most suitable for *P*. *papatasi* occurrence and the suitability range increases with the progression of the warmer summer months. Suitable areas for CL vector occurrence coincide with highly populated parts of the country. Geographically targeting mitigation strategies can benefit a large proportion of the population at risk of CL, and thus promote resource-efficient interventions. This is, however, one component within the complex requirements for the implementation of a comprehensive national strategy for CL prevention and control in Jordan. In addition to the epidemiological elements, other socio-economic and logistical constraints will determine the success of a national vector control program.
